# Komplementärmedizinische Therapieansätze bei krebsbedingter Fatigue

**DOI:** 10.1007/s00103-024-03957-8

**Published:** 2024-10-07

**Authors:** Alina Busch, Alena Krause, Matthias Rostock

**Affiliations:** Universitäres Cancer Center Hamburg, II. Medizinische Klinik und Poliklinik (Onkologie, Hämatologie, Knochenmarktransplantation mit Sektion Pneumologie), Universitätsklinikum Hamburg Eppendorf, Martinistraße 52, 20246 Hamburg, Deutschland

**Keywords:** Mind-Body-Medizin, Phytotherapie, Yoga, Akupunktur, Leitlinienempfehlungen, Mind-body medicine, Herbal medicine, Yoga, Acupuncture, Guideline recommendations

## Abstract

Ein Großteil der Krebspatient:innen erlebt im Verlauf ihrer Erkrankung inadäquate Erschöpfungszustände (Fatigue). Krebsbedingte Fatigue (Cancer-related Fatigue – CRF) ist ein multidimensionaler Symptomkomplex, der durch eine Vielzahl von Faktoren beeinflusst wird. Komplementärmedizinische Ansätze bieten potenziell vielversprechende Strategien, um die Erschöpfung zu adressieren und können daher eine wertvolle Ergänzung zu den konventionellen Therapieverfahren darstellen.

In dieser narrativen Übersichtsarbeit werden komplementärmedizinische Therapieansätze bei krebsbedingter Fatigue entsprechend der historischen Entwicklung und der aktuellen wissenschaftlichen Evidenz dargestellt. Dabei liegt der Schwerpunkt auf den Methoden mit der aktuell höchsten Evidenz, in Orientierung an den Empfehlungen nationaler und internationaler Leitlinien. Therapieverfahren der Mind-Body-Medizin wie achtsamkeitsbasierte Stressreduktion (MBSR) und achtsamkeitsbasierte kognitive Therapie (MBCT), Yoga, Tai-Chi und Qigong sowie Akupunktur, Akupressur, Moxibustion und phytotherapeutische Behandlungsansätze werden vorgestellt.

In Deutschland gilt für die hier aufgeführten komplementärmedizinischen Therapieverfahren in der Regel keine Leistungsgewährung durch die Krankenkassen, wenn auch einige wenige Kliniken komplexe Programme aus der Mind-Body-Medizin entwickelt haben, für die bereits eine Kostenübernahme erreicht werden konnte. Eine komplementärmedizinische Behandlung der krebsbedingten Fatigue, die den Empfehlungen nationaler und internationaler Leitlinien entspricht, kann daher in Deutschland in der Regel nur als Privatleistung oder im Rahmen von Studien durchgeführt werden.

## Abstract (en)

The majority of cancer patients experience fatigue during the course of their illness. Fatigue should not be seen as an inevitable consequence of cancer and its treatment. Cancer-related fatigue (CRF) is a multidimensional symptom complex that is influenced by a variety of factors. Complementary medicine approaches offer potentially promising strategies to address this fatigue and can therefore be a valuable addition to conventional therapies.

In this narrative review, complementary medicine treatment approaches for tumor-associated fatigue are presented according to the historical development and current scientific evidence. The focus is on methods with the highest current evidence based on the recommendations of national and international guidelines. Therapeutic approaches from mind-body medicine, such as mindfulness-based stress reduction (MBSR), mindfulness-based cognitive therapy (MBCT), yoga, tai chi, and qigong, as well as acupuncture, acupressure, moxibustion, and phytotherapeutic treatment approaches are presented.

In Germany, the complementary therapies listed here are not generally covered by health insurances. However, a few clinics have developed comprehensive programs in mind-body medicine, for which cost coverage has already been obtained. As a result, complementary medical treatments for cancer-related fatigue that adhere to national and international guidelines are typically available only as private services or within the framework of study participation.

## Hintergrund

Fatigue ist ein multidimensionaler Symptomkomplex, der durch eine Vielzahl von Faktoren beeinflusst wird. Der vorliegende Artikel fokussiert sich auf komplementärmedizinische Behandlungsmethoden bei krebsbedingter Fatigue (engl. *Cancer-related Fatigue,* CRF). Klinische Erfahrung und Studium der wissenschaftlichen Literatur legen aber eine weitgehende Übertragbarkeit auf Fatigue im Kontext weiterer Therapiesituationen nahe.

CRF ist ein Symptomkomplex, der die Lebensqualität erheblich einschränkt und sowohl physische, emotionale als auch kognitive Aspekte umfasst. Schwere Fatigue kann die Durchführbarkeit tumorgerichteter Therapien beeinträchtigen und hierüber die Prognose beeinflussen [1]. So war CRF in einer Registerstudie mit einer erhöhten Sterblichkeit assoziiert [2]. CRF kann Personen mit einer Krebserkrankung in jedem Stadium und zu jedem Zeitpunkt im Krankheitsverlauf betreffen. Eine nationale Querschnittserhebung in den USA berichtet, dass bis zu 80 % der Patient:innen, die sich einer antitumoralen Therapie unterziehen, an CRF leiden [3]. Eine kürzlich durchgeführte Metaanalyse berichtet Prävalenzen von CRF zwischen 11 % und 99 % [4]. Die aggregierte Prävalenz wurde von den Autor:innen einer aktuellen Metaanalyse von 84 Beobachtungsstudien auf 52 % geschätzt [5, 6].

Fatigue kann als erstes und einziges Symptom einer Tumorerkrankung auch bereits Monate vor der Diagnosestellung auftreten und wird als das am längsten anhaltende und am stärksten belastende Symptom nach Abschluss der Behandlung empfunden, das sogar bei ansonsten völlig gesunden Krebsüberlebenden und auch noch länger als 5 Jahre nach abgeschlossener Therapie persistieren kann [7–10].

CRF ist selten ein isoliertes Symptom; es tritt meist in Verbindung mit anderen somatischen und psychischen Beschwerden auf. Die Ursachen sind multifaktoriell und die zugrunde liegenden pathophysiologischen Mechanismen sind nicht vollständig verstanden. Es wird angenommen, dass sowohl verschiedene Veränderungen auf Zellebene und in Regelkreisläufen als auch psychosoziale Faktoren (Selbstwirksamkeit, Kausalattributionen, Erwartungen, Bewältigung und soziale Unterstützung) eine Rolle spielen und sich gegenseitig beeinflussen können [11–19]. Diese Aspekte werden in weiteren Beiträgen dieses Themenheftes des Bundesgesundheitsblatts ausführlich dargelegt.

Die weitverbreitete Fehlannahme, dass Müdigkeit und körperliche Schwäche unvermeidliche Begleiterscheinungen einer Krebserkrankung und deren Behandlung sind, bleibt ein häufiges Problem in diesem Kontext. Unter anderem hierdurch erhält nur ein Viertel der von CRF betroffenen Patient:innen diesbezügliche Therapieempfehlungen [11].

Komplementärmedizinische Behandlungsansätze umfassen eine Vielzahl von Methoden, die in Ergänzung zu konventionellen medizinischen Therapien angewendet werden können. Zu den konventionellen Verfahren zählen kognitive Verhaltenstherapie, Psychoedukation und Sport- und Bewegungsinterventionen, die an anderer Stelle im Themenheft näher beschrieben werden. Wichtige komplementärmedizinische Ansätze, die in dem vorliegenden Artikel vorgestellt werden, sind Mind-Body-Medizin-Interventionen, wie achtsamkeitsbasierte Stressreduktion (MBSR), achtsamkeitsbasierte kognitive Therapie (MBCT), Yoga, Tai-Chi und Qigong, sowie Akupressur und Akupunktur, einschließlich Moxibustion, und die Phytotherapie.

Die Behandlung des Symptomkomplexes Fatigue ist Gegenstand der S3-Leitlinie für Komplementärmedizin in der Behandlung onkologischer Patient:innen [20]. Ebenso enthalten die Leitlinien der Europäischen Gesellschaft für Internistische Onkologie (*European Society of Medical Oncology* – ESMO) und des *National Comprehensive Cancer Center* (NCCN) Empfehlungen zur komplementärmedizinischen Behandlung von krebsbedingter Fatigue [21, 22]. Mitte Mai 2024 veröffentlichte die *American Society for Clinical Oncology* (ASCO) gemeinsam mit der *Society for Integrative Oncology* (SIO) ihre aktualisierte Leitlinie zu tumorbedingter Fatigue bei Krebsüberlebenden, welche erstmals auch Empfehlungen für Patient:innen unter Therapie beinhaltet [23].

Grundsätzlich sollte sich die Auswahl geeigneter komplementärmedizinischer Therapieverfahren immer auf die 3 Säulen der evidenzbasierten Medizin nach Sackett beziehen und damit neben der externen Evidenz v. a. aus randomisierten kontrollierten Studien (RCTs), systematischen Reviews und Metaanalysen auch die klinische Expertise des/der Behandler:in sowie die Patient:innenpräferenzen einbeziehen [24]. Dabei sind nicht nur Erkrankungssituation und Verlauf der Erkrankung von Bedeutung, sondern auch Begleiterkrankungen und weitere bestehende Symptome.

Im Folgenden wird ein Überblick über komplementärmedizinische Behandlungsansätze gegeben, deren Wirksamkeit durch RCTs nachgewiesen werden konnte, sowie über vielversprechende Therapieansätze, die weiterführender Untersuchungen bedürfen. Es ist wichtig zu betonen, dass die vorliegenden randomisierten kontrollierten Studien zu einem großen Anteil mit Patientinnen mit Mammakarzinom durchgeführt wurden [25], was die allgemeine Übertragbarkeit der Ergebnisse einschränken kann.

## Mind-Body-Medizin

Psychosoziale Interventionen bei CRF adressieren sowohl die psychischen und emotionalen Aspekte als auch die sozialen Umstände und Beziehungen von Krebspatient:innen. Die Interventionsmöglichkeiten sind hierbei vielfältig. Ein wichtiges komplementärmedizinisches Konzept im Kontext der psychosozialen Interventionen bei CRF ist die „Mind-Body-Medizin“ (MBM). In den 1970er-Jahren führte Herbert Benson den Ansatz der Mind-Body-Medizin an der Harvard Medical School ein und gründete dort später das erste Institut für diesen Bereich [26]. Er prägte den wissenschaftlichen Begriff *Relaxation Response*, um die durch Meditation ausgelöste Umkehrung der physiologischen Stressreaktion zu beschreiben, als Gegensatz zu dem früher durch Walter Cannon beschriebenen *Fight-or-Flight*-Konzept [27, 28]. Benson war einer der ersten westlichen Mediziner, der Aspekte der Spiritualität, Religiosität und traditionellen Heilkünste in konventionelle Therapieansätze integrierte [29].

Das Konzept der Mind-Body-Medizin strebt im Sinne einer individuellen gesundheitsfördernden, salutogenetischen Medizin danach, über eine Interaktion von Körper, Gehirn, Geist und Verhalten Selbstheilungspotenziale zu trainieren und zu stärken [29, 30]. In einem multimodalen Konzept werden psychologische Techniken mit körperlichen Praktiken kombiniert, um Stress abzubauen, Entspannung und Gesundheit zu fördern und die psychische Resilienz durch Selbstregulation zu stärken.

Die zuvor genannten Leitlinien empfehlen verschiedene Mind-Body-Medizin-Interventionen zur Behandlung von CRF.

### Achtsamkeitsbasierte Stressreduktion (MBSR)

Unter den achtsamkeitsbasierten klinischen Interventionen werden die „achtsamkeitsbasierte Stressreduktion“ (Mindfulness-based Stress Reduction – MBSR) und die „achtsamkeitsbasierte kognitive Therapie“ (MBCT, siehe unten) in der Onkologie am häufigsten eingesetzt [21]. Auch Achtsamkeitsmeditation, die ihre Wurzeln im Buddhismus hat, zeigt Wirksamkeit, jedoch ist die Datenlage hierfür nicht so solide wie für MBSR [23, 31].

MBSR ist ein strukturiertes Gruppentherapieprogramm, das in den späten 1970er-Jahren von Jon Kabat-Zinn an der Massachusetts Medical School konzipiert wurde. Es wird über 8 Wochen von speziell ausgebildeten Therapeut:innen angeleitet. Das Programm umfasst wöchentlich stattfindende Gruppensitzungen á 2,5 h und in der Regel einen 6‑ bis 7‑stündigen Tag in Stille zwischen dem 6. und 7. Kurstreffen. Achtsamkeitsmeditationen wie Bodyscan, achtsame Bewegungen und Sitzmeditation werden in Gruppensitzungen angeleitet und in täglicher Praxis mit Audioanleitungen eigenständig geübt. Neben dem Austausch in der Gruppe über die Erfahrungen mit der Meditation werden Schwerpunktthemen wie Wahrnehmung, Umgang mit Stress und achtsame Kommunikation erarbeitet und weiteres Lehrmaterial zur Verfügung gestellt. MBSR fördert die Selbstwahrnehmung und eine nicht wertende Akzeptanz von gegenwärtigen Erfahrungen, wodurch Stress reduziert und die emotionale Reaktionsfähigkeit reguliert und verbessert wird [32]. Eine signifikante Verringerung von Fatigue, Schlafstörungen und Stress wurde bei ambulanten onkologischen Patient:innen nachgewiesen [32, 33]. Dieser Effekt hielt auch über einen Zeitraum von 6 Monaten nach Abschluss der Intervention an [32]. Eine randomisierte kontrollierte Studie (RCT) aus den USA mit 167 Patient:innen nach einer Brustkrebsbehandlung bestätigte den anhaltenden Effekt zumindest 6 Wochen nach Abschluss der Intervention [34].

Eine multinationale Studie aus den Niederlanden untersuchte den Effekt einer 12-wöchigen regelmäßigen Nutzung der Gesundheitsapplikation „Untire“ auf Fatigue bei Krebspatient:innen sowohl während als auch nach abgeschlossener Therapie. Die Applikation leitete die Patient:innen in der Interventionsgruppe (*n* = 545) zu Übungen in achtsamkeitsbasierter Stressreduktion, aber auch zu gesteigerter physischer Aktivität, Psychoedukation und Selbstwirksamkeit an [35]. Es konnte ein signifikanter Effekt auf die CRF nachgewiesen werden [35].

### Achtsamkeitsbasierte kognitive Therapie (MBCT)

Das Programm der achtsamkeitsbasierten kognitiven Therapie (Mindfulness-based Cognitive Therapy –MBCT) wurde von den klinischen Psychologen Zindel Segal, Mark Williams und John Teasdale entwickelt. Initial standen die Grundprinzipien und die Wirksamkeit von MBCT zur Rückfallprävention bei Depressionen im Vordergrund [36]. Das 8‑wöchige Konzept vermittelt in wöchentlichen Gruppensitzungen Strategien aus der kognitiven Verhaltenstherapie mit Elementen der achtsamkeitsbasierten Stressreduktion nach Kabat-Zinn. Durch die Integration von Achtsamkeitspraktiken und kognitiven Therapieansätzen zielt MBCT darauf ab, das Bewusstsein für den gegenwärtigen Moment zu stärken und dysfunktionale Denkmuster zu identifizieren und zu transformieren. Es wird hierbei eine „dezentrierte Perspektive gegenüber dem Erleben gefördert, bei der negative Gedanken oder Gefühle als Ereignisse im Geist betrachtet werden, nicht als Teil des Selbst oder zwangsläufig als wahr“ [36]. Mittlerweile wird das Konzept indikationsübergreifend auch außerhalb von depressiven Störungen angewendet und zeigt in der Therapie der CRF vielversprechende Ergebnisse:

In einer niederländischen RCT zeigte MBCT in der Interventionsgruppe von 59 Krebsüberlebenden sowohl direkt nach Abschluss der Intervention als auch 6 Monate später eine signifikante Reduktion der CRF [37]. Zwei weitere niederländische Studien bestätigen das Ergebnis einer signifikanten Reduktion der Fatigue bei Krebspatient:innen auch nach einer 8‑wöchigen webbasierten MBCT-Intervention [38, 39].

### Yoga

Als eine jahrtausendealte Praxis wurzelt Yoga in den religiösen und philosophischen Traditionen Indiens. Seit den 1960er-Jahren hat Yoga im Westen zunehmend an Popularität gewonnen. Die traditionelle Praxis des Yoga basiert u. a. auf den Yogasutras von Maharishi Patañjali [40]. Im therapeutischen Kontext wird häufig die Form des „Hatha-Yoga“ angeleitet.

Lehrer:innen sollten eine spezielle Zusatzausbildung für den Umgang mit Krebspatient:innen haben. Diese beschränkt sich jedoch nicht selten nur auf einen unstandardisierten 40-stündigen Kurs ohne festgelegtes Curriculum. Auch die Art der Yogaintervention, die Interventionsdauer und die Wochenstundenzeit variieren erheblich in den vorhandenen RCTs. Die Richtlinie CLARIFY (Checklist Standardising the Reporting of Interventions for Yoga) dient deshalb seit 2022 als eine speziell auf Yoga ausgerichtete Berichterstattungsrichtlinie, die bei randomisierten kontrollierten Studien, Beobachtungsstudien und Fallberichten herangezogen werden kann [41]. Alle genannten Leitlinien empfehlen Yoga bei CRF. Die ASCO beschränkt ihre Empfehlung auf die Zeit nach der tumorspezifischen Therapie wegen einer unzureichenden Studienlage während der Behandlung. Die NCCN-Leitlinie empfiehlt Yoga explizit auch während der Therapie.

Hervorzuheben ist erneut, dass die meisten Studien zu Yoga und Fatigue mit Brustkrebspatient:innen durchgeführt wurden (Anteil bis 76 %; [42]). Eine US-amerikanische multizentrische RCT untersuchte ein standardisiertes Yogaprogramm für Krebsüberlebende (YOCAS). Die Studie zeigte nach nur 4 Wochen Training mit sanftem „Hatha-Yoga“ und „Restorative-Yoga“ eine signifikante Reduktion der Fatigue in der Interventionsgruppe (*n* = 177), die 2‑mal wöchentlich 75-minütige Treffen mit Anleitung zu Asanas, Pranayama und Achtsamkeitsmeditation umfasste. Die Therapieadhärenz in dieser Studie war hoch: Teilnehmer:innen (95 % weiblich) praktizierten durch zusätzliche Eigenübung durchschnittlich mehr als die empfohlene Mindestzeit [43]. Zum Vergleich: In einer deutschen Studie, die keinen Vorteil für eine Yogaintervention bei 54 Patient:innen (61 % männlich) mit kolorektalen Karzinomen zeigen konnte, lag die Teilnahmerate der Patient:innen an einem 10-wöchigen Programm bei nur ca. 50 % [44].

Eine weitere Studie mit 100 Brustkrebspatient:innen im Interventionsarm zeigte eine Reduktion der Fatigue direkt nach einer 12-wöchigen Hatha-Yogaintervention und auch noch 3 Monate danach [45]. Eine weitere RCT mit 60 Brustkrebsüberlebenden in Taiwan zeigte im Interventionsarm eine signifikante Reduktion der Fatigue nach einem 8‑wöchigen Yogaprogramm, das aus einer 60-minütigen Einheit des „Anusara-Yoga“ mit zusätzlichen sanften Dehnungs- und Entspannungseinheiten 2‑mal pro Woche bestand [46]. Eine Metaanalyse ergab, dass innerhalb der Yogainterventionen keine spezifischen Kursmerkmale vorteilhafter waren. Der zeitliche Rahmen der Intervention hatte insbesondere keinen Einfluss auf die Effektstärke [42]. Die Wirksamkeit einer Yogaintervention hängt offenbar stark von der Adhärenz der Teilnehmer:innen und einer regelmäßigen Eigenpraxis ab.

### Qigong und Tai-Chi

Als Teil der traditionellen chinesischen Bewegungskünste haben Qigong und Tai-Chi eine jahrhundertealte Geschichte. Tai-Chi entstand als meditative Kampfkunst und besteht aus langsamen, fließenden Bewegungen, die mit kontrollierter Atmung und Meditation kombiniert werden. Qigong hat seine Wurzeln in daoistischen, buddhistischen und konfuzianischen Gesundheitspraktiken und konzentriert sich traditionell durch einfache, repetitive Bewegungen mit Atemübungen und Meditation auf den Fluss von Lebensenergie (Qi) im Körper.

Eine RCT mit 87 Brustkrebsüberlebenden zeigte eine signifikante Reduktion der CRF nach 12-wöchiger Qigong- und Tai-Chi-Praxis, sowohl direkt nach der Intervention als auch nach einem 3‑monatigen Intervall [47]. Eine weitere RCT zeigte einen ähnlichen Effekt für Tai-Chi bei älteren Krebspatient:innen mit verschiedenen Krebsarten [48]. Eine RCT aus Taiwan berichtete von einem positiven Effekt einer 3‑wöchigen Qigongintervention bei Hodgkin-Lymphom-Patient:innen unter Chemotherapie [49].

Zwei RCTs aus China zeigten positive Effekte für Qigong bei Patient:innen mit kolorektalem Karzinom und Tai-Chi bei Nasopharynxkarzinom-Patient:innen unter Chemo- bzw. Radiochemotherapie [50, 51]. Eine weitere multizentrische RCT zu Qigong bei CRF läuft derzeit in Australien und Malaysia [52].

## Akupunktur, Akupressur und Moxibustion

Zentrale Praktiken der Traditionellen Chinesischen Medizin (TCM) sind Akupunktur, Akupressur und Moxibustion. Sie basieren auf dem traditionellen Verständnis, dass Krankheiten mit einer Störung der Lebensenergie (Qi) im Körper einhergehen und der gestörte Energiefluss durch Punktieren (Akupunktur), Erwärmen (Moxibustion) oder Massieren bzw. Drücken (Akupressur) spezifischer Punkte entlang der energetischen Meridiane des Körpers wiederhergestellt werden kann [53].

Die NCCN empfiehlt Akupunktur bei CRF, während die ASCO und die deutsche Leitlinie dies als optionale Maßnahme betrachten. Die ASCO bemängelt unzureichende Daten zur endgültigen Beurteilung der Wirksamkeit. Die Autor:innen einer aktuellen chinesischen Metaanalyse werteten die Ergebnisse von 34 RCTs aus den letzten 40 Jahren zur Akupunktur bei CRF als positiven Einfluss [54], wobei die Behandlungen der unterschiedlichen Studienpopulationen nachvollziehbarerweise erhebliche Unterschiede in Bezug auf die Stimulationspunkte, die Anwendungsdauer und die Evaluation der Fatigue aufwiesen. Die Akupunkturpunkte ST36 (Magenmeridian), KI3, SP6 (konvergierender Akupunkturpunkt der Leber‑, Milz- und Nieren-Yin-Kanäle) und energieassoziierte Punkte einschließlich LI4 und DU20 können aus Sicht der Autor:innen hierbei als die wichtigsten Akupunkturpunktkombinationen zur Behandlung von CRF angesehen werden [54]. Zwei weitere Metaanalysen bestätigten einen positiven Einfluss von Akupunktur auf CRF [55]. Diese Annahmen werden auch von einem aktuellen systematischen Review gestützt, der eine Zusammenfassung verschiedener systematischer Reviews über komplementäre Therapieansätze bei CRF bietet [25].

Aurikuläre Akupressur (Akupressur von Ohrakupunkturpunkten) zeigte bei Lungenkrebspatient:innen unter Chemotherapie eine Linderung der CRF in einer RCT [56].

Eine große RCT von Zick et al. zeigte, dass tägliche Akupressur, bei der Brustkrebsüberlebende über 6 Wochen hinweg für 30 min festgelegte Kombinationen von Akupunkturpunkten selbst massierten, die krebsbedingte Erschöpfung signifikant verbesserte [57]. Bei mehr als 60 % der Teilnehmerinnen normalisierte sich die Fatigue nach Abschluss der Intervention (Vergleichsgruppe 31,3 %.). Die Gruppe, die entspannungsfördernde Punkte behandelte, zeigte zusätzlich eine Verbesserung von Schlaf- und Lebensqualität. Die dazugehörige Pilotstudie, die mit Krebspatient:innen mit gynäkologischen Karzinomerkrankungen durchgeführt worden war, hatte sowohl bei Akupressur von stimulierenden Akupunkturpunkten als auch bei Akupressur von relaxierenden Akupunkturpunkten zu einer signifikanten Reduktion der CRF-Beschwerden geführt. Besonders interessant in dieser Studie war, dass der Therapieeffekt bei der Anwendung von Akupressur an relaxierenden Akupunkturpunkten noch einmal signifikant stärker war als bei der Patient:innengruppe, die mit stimulierenden Akupunkturpunkten arbeitete und ebenfalls profitierte [58]. Dieses Studiendesign, mit dem Patient:innen unter Anleitung in die Lage versetzt werden, sich selbst zu behandeln, ist im Hinblick auf Selbstwirksamkeit, Wirtschaftlichkeit und eine Übertragbarkeit auf die Anwendung außerhalb von großen Versorgungszentren von nicht unerheblicher Bedeutung.

Die ASCO bewertet die aktuelle Datenlage zu Moxibustion mit einer bedingten Empfehlung, basierend auf 2 positiven randomisierten kontrollierten Studien mit insgesamt 174 Patient:innen [59, 60]. Eine aktuelle Metaanalyse aus 24 RCTs zu Moxibustion und CRF bestätigte den signifikanten Einfluss der Intervention [61]. Eine kürzlich publizierte RCT zu Infrarotlaser-Moxibustion (*n* = 56) und CRF bei Brustkrebsüberlebenden zeigte eine signifikante Reduktion der CRF im Vergleich zur Wartegruppe, ohne dass ebenfalls eine statistische Signifikanz gegenüber der Sham-Kontrolle bestätigt werden konnte [62].

## Phytotherapie

Die Vorgehensweisen in der onkologischen Therapie werden zunehmend individueller und dadurch oft effektiver in der Behandlung von Krankheiten, die auf spezifische Pathomechanismen bezogen werden. Im Gegensatz zu solchen immer selektiveren Therapieansätzen, wie beispielsweise in der personalisierten Onkologie, haben phytotherapeutische Extrakte mit ihrem breiten Spektrum an Wirkstoffen und entsprechend breitem Wirkprofil in der Regel pleiotrope Effekte, d. h. Wirkungen auf mehrere Zielstrukturen. Bei einem multifaktoriellen Symptomkomplex wie der CRF erscheint ein solcher Ansatz besonders sinnvoll. Bisher haben lediglich Zubereitungen aus Ginsengwurzel in verschiedenen Leitlinien eine bedingte Empfehlung erhalten. Vor diesem Hintergrund werden einige wenige Heilpflanzen zusätzlich vorgestellt, die in dieser Indikation häufige Verwendung finden und für die wissenschaftliche Daten aus klinischen Studien zu Fatigue vorliegen.

In der Phytotherapie werden seit Langem bei chronischen Erschöpfungszuständen Heilpflanzen mit adaptogenen Eigenschaften eingesetzt. Adaptogene sind Präparate, die einen Organismus gegenüber physikalischem, chemischem, biologischem und psychischem Stress widerstandsfähiger machen, das heißt die Adaptation und Toleranz gegenüber Stressoren erhöhen [63]. Derzeit wichtige Vertreter sind unter anderen Ginseng (*Panax ginseng *und *Panax quinquefolius) *sowie Rosenwurz (*Rhodiola rosea). *Weitere in der Behandlung von CRF häufig angewandte Phytotherapeutika sind die weißbeerige Mistel (*Viscum album L.*) und der Baldrian (*Valeriana officinalis L.*).

### *Panax ginseng* und *Panax quinquefolius *(Ginseng).

Zubereitungen aus der Wurzel von *Panax ginseng *werden seit vielen Jahrhunderten in der TCM und anderen asiatischen Medizinsystemen u. a. bei Erschöpfungszuständen eingesetzt. Der Einsatz erfolgt mit dem Ziel, den menschlichen Organismus darin zu unterstützen, sich erhöhten körperlichen und emotionalen Anforderungen besser anpassen zu können. Vergleichbar werden mittlerweile auch Zubereitungen aus der Wurzel des amerikanischen Ginsengs *(Panax quinquefolius*) angewandt. Es steht eine ganze Reihe von Ginsengwurzelextrakten in Form von Nahrungsergänzungsmitteln und pflanzlichen Arzneimitteln zur Verfügung. Die heute verwendeten Wirkstoffdarreichungen weisen i. d. R. einen Ginsenosid-Gehalt von 3–7 % auf. Eine systematische Übersichtsarbeit von 2021 kam zu dem Schluss, dass mit der Einnahme von Ginsengwurzel Fatiguebeschwerden bei Krebspatient:innen unter antitumoraler Behandlung signifikant gesenkt werden können [64]. Die aktuelle ASCO-SIO-Guideline empfiehlt eine Dosierung von mindestens 2000 mg Ginsengwurzel täglich bei einer Einnahmedauer von mindestens 8 Wochen [23]. Sowohl für den amerikanischen als auch für den asiatischen Ginseng wurde unter diesen Voraussetzungen eine Besserung von Beschwerden unter CRF beobachtet, diese wurden aber primär in der begleitenden Behandlung zur Chemotherapie und bislang nicht im Anschluss an die antitumorale Behandlung erhoben.

### *Rhodiola rosea L.* (Rosenwurz).

Extrakte aus der Wurzel von *Rhodiola rosea L.* werden seit vielen Jahren als Tonikum bei Erschöpfungszuständen und in den letzten Jahrzehnten auch als Adaptogen bei Fatigue und krebsbedingter Fatigue eingesetzt. Die Anwendung ist vor allem in den osteuropäischen Staaten und in Skandinavien sowie zunehmend in den deutschsprachigen Ländern bekannt. Gemäß der Monographie des Ausschusses für pflanzliche Arzneimittel (*Committee on Herbal Medicinal Products *(HMPC)) der Europäischen Arzneimittelagentur (*European Medicines Agency* (EMA)) zur traditionellen Anwendung können Extrakte aus dem Wurzelstock der Rosenwurz zur Linderung von Stresssymptomen wie Müdigkeit und Schwächegefühlen eingesetzt werden [65]. Bis 2021 wurden eine Reihe von RCTs durchgeführt, die Hinweise auf eine mögliche therapeutische Wirksamkeit zur Beeinflussung von Beschwerden durch Fatigue sowie der körperlichen und geistigen Leistungsfähigkeit als auch des psychischen Befindens liefern. Rosenwurzextrakt ist ein Vielstoffgemisch, das u. a. Phenylpropanoide (u. a. Rosavin) und Phenylethanoide (u. a. Salidrosid) enthält. Die Einnahme erfolgt oral, in der Regel mit einer täglichen Dosis von 200–600 mg Extrakt, aufgeteilt in eine morgendliche und mittägliche Einnahme.

### *Viscum album L.* (weißbeerige Mistel).

Die Mistel ist ein Halbparasit, der auf verschiedenen Bäumen wächst. Die therapeutische Nutzung ist seit Jahrhunderten bekannt. Die Anwendung erfolgt als subkutan zu verabreichendes Injektionspräparat v. a. im deutschen Sprachraum, aber auch in vielen weiteren Ländern weltweit. In der deutschen Leitlinie für Komplementärmedizin in der Behandlung von onkologischen Patient:innen hat die Mistel, neben 3 weiteren Heilpflanzen, eine positive Bewertung der Expertenkommission erhalten: Basierend auf den Ergebnissen vorliegender randomisierter Therapiestudien kann die Anwendung der Mistel gemäß Leitlinie zur Verbesserung der Lebensqualität erwogen werden [20].

Bezüglich CRF hat eine Metaanalyse von 2022, die 12 RCTs mit 1494 Patient:innen umfasste, gezeigt, dass eine Behandlung mit Mistelextrakten einen moderaten Effekt auf CRF hat, den die Autor:innen als vergleichbar mit dem von körperlicher Aktivität einschätzten [66]. Wichtig ist, dass in sämtlichen RCTs die Fatigue nicht als primärer Zielparameter untersucht worden ist. Entsprechend sollten diese Ergebnisse in größeren randomisierten Studien mit Mistelgesamtextrakt als primärem Zielparameter überprüft werden.

### *Valeriana officinalis L.* (Baldrian).

In einer umfangreichen randomisierten, doppelblinden Phase-III-Studie in den USA erhielten 227 Krebspatient:innen mit Schlafstörungen entweder über einen Zeitraum von 8 Wochen 450 mg Baldrianwurzelextrakt oder ein Placebo, das eine Stunde vor dem Schlafengehen oral verabreicht wurde. Der primäre Hauptzielparameter, die Schlafstörungen, gemessen mit dem *Pittsburgh Sleep Quality Index,* verbesserte sich in Therapie- und Kontrollgruppe gleichermaßen ohne signifikanten Unterschied. Aber der sekundäre Zielparameter, die Beschwerden durch CRF, gemessen mit dem *Brief Fatigue Inventory* (BFI), besserte sich in der Verumgruppe signifikant gegenüber Placebo [67].

Diese Ergebnisse sind in Anwendung und Behandlungsergebnissen mit den Ergebnissen der randomisierten Akupressurstudie (s. oben) vergleichbar, in der die Behandlung von entspannungsfördernden Akupunkturpunkten ein signifikant besseres Ergebnis erbracht hatte als die Behandlung der stimulierenden Akupunkturpunkte [58]. Und sie korrespondieren ebenso mit den sehr überzeugenden Daten aus den RCTs mit MBSR, Tai-Chi und Qigong bei dieser Indikation. Ganz offensichtlich sind für einen Großteil der Patient:innen therapeutische Maßnahmen, die Entspannungs- und Regenerationsförderung im Fokus haben, besonders hilfreich.

### Potenzial von phytotherapeutischen Ansätzen

Die Phytotherapie bietet neben den hier abgehandelten Beispielen, für die mehrheitlich entsprechend einer Reihe von RCTs CRF-bezogene Symptombesserungen gezeigt wurden, weitere Therapiemöglichkeiten, für die allerdings derzeit keine CRF-spezifischen RCTs vorliegen. Allerdings spricht einiges dafür, dass zumindest Teile der Pathophysiologien in diesen Studien mit denen der CRF vergleichbar sind, sodass eine Übertragbarkeit von Behandlungsergebnissen möglich erscheint.

Die krebsbedingte Fatigue kann sich bei den Betroffenen individuell vor einem sehr unterschiedlichen Hintergrund entwickeln und durch stark variierende Begleitfaktoren gefördert werden. Dies können soziale Mediatoren sein, psychische Begleitbeschwerden wie Ängste, Anspannungszustände oder Depressionen oder körperliche Begleitbeschwerden, wie z. B. Ernährungsstörungen oder endokrinologische Fehlfunktionen. Bei manifesten Krankheitsbildern würden diese Beschwerden eine spezifische medizinische Intervention erfordern. Bei subsyndromalen Beeinträchtigungen wäre diese nicht zwangsläufig erforderlich, aber eine phytotherapeutische Behandlung möglicherweise durchaus hilfreich.

## Leitlinienempfehlungen

Tab. 1 bietet einen Überblick über die Empfehlungen aus den aktuellen Leitlinien zur Komplementärmedizin in der Behandlung von onkologischen Patient:innen der Deutschen Krebsgesellschaft (DKG) und der Arbeitsgemeinschaft der Wissenschaftlichen Medizinischen Fachgesellschaften (AWMF), des *National Comprehensive Cancer Network*, der *European Society for Medical Oncology* sowie der *American Society of Clinical Oncology* zur Behandlung der *Cancer-related Fatigue*. Mit dem Ziel, einen noch umfassenderen Überblick geben zu können, finden sich in dieser Tabelle auch nichtkomplementäre Verfahren wie die psychoedukative Therapie und die kognitive Verhaltenstherapie sowie körperliche Aktivität und Sport. Zusätzlich sind hier auch Therapieverfahren beschrieben, von denen explizit abgeraten wird oder zu denen sehr heterogene Beurteilungen vorliegen.

**Tab. 1:** Leitlinienempfehlungen zu komplementärmedizinischen und supportiv medizinischen Therapieverfahren bei onkologischen Patient:innen mit tumorbedingter Fatigue (CRF)

	S3-Leitlinie Komplementärmedizin Onkologie [20]	NCCN CRF [22]	ESMO CRF [21]	ASCO-SIO CRF [23]
**Supportivtherapie**				
**Körperliche Aktivität und Sport**	Empfehlungsgrad A: starke Empfehlung „soll“	Empfehlung (Kategorie 1) für Patient:innen unter Therapie und nach Abschluss der Therapie	Mäßige Empfehlung für nichtkachektische Patient:innen (I, B)	Starke Empfehlung (moderate Evidenz) unter und nach Therapie
**Kognitive Verhaltenstherapie**	–	Empfehlung (Kategorie 1) für Patient:innen unter Therapie und nach Abschluss der Therapie	Mäßige Empfehlung (II, B)	Starke Empfehlung (moderate Evidenz) unter und nach Therapie
**Psychoedukative Therapie**	–	Empfehlung (Kategorie 1) für Patient:innen unter Therapie und nach Abschluss der Therapie	Mäßige Empfehlung (II, B)	Bedingte Empfehlung (moderate Evidenz) unter Therapie
**Ernährungsberatung**	–	Empfehlung (Kategorie 2A) für Patient:innen unter Therapie und nach Abschluss der Therapie	–	–
**Achtsamkeitsbasierte Interventionen**				
**Mindfulness-based Stress Reduction (MBSR)**	Empfehlungsgrad 0: Empfehlung offen „kann“ nach adjuvanter Therapie	Empfehlung (Kategorie 1) für Patient:innen nach Abschluss der Therapie	Geringfügige Empfehlung (II, C)	Starke Empfehlung (moderate Evidenz) unter und nach Therapie für MBSR und MBCT
**Yoga**	Empfehlungsgrad B: Empfehlung „sollte“ während und nach Abschluss von Chemo‑/Radiotherapie	Empfehlung (Kategorie 1) für Patient:innen unter Therapie und nach Abschluss der Therapie	Geringfügige Empfehlung (II, C) nach Abschluss der Therapie	Bedingte Empfehlung (niedrige Evidenz) nach Therapie; keine ausreichende Evidenz für Empfehlung unter Therapie
**Qigong/Tai-Chi**	Empfehlungsgrad B: Empfehlung „sollte“ während und nach Abschluss von Chemo‑/Radiotherapie	Diskussion der vorliegenden Studien ohne Empfehlungsabgabe für oder wider	–	Starke Empfehlung (moderate Evidenz) unter Therapie, keine ausreichende Evidenz für oder gegen Empfehlung nach Therapie
**Traditionelle Chinesische Medizin**				
**Akupunktur**	Empfehlungsgrad 0: Empfehlung offen „kann“	Empfehlung (Kategorie 2A) für Patient:innen unter Therapie und nach Abschluss der Therapie	Das Gremium hat keinen Konsens erzielt	Bedingte Empfehlung (niedrige Evidenz) unter Therapie, keine ausreichende Evidenz für oder gegen Empfehlung nach Therapie
**Akupressur**	Empfehlungsgrad 0: Empfehlung offen „kann“	Diskussion der vorliegenden Studien ohne Empfehlungsabgabe für oder wider	–	Bedingte Empfehlung (niedrige Evidenz) nach Therapie, keine ausreichende Evidenz für oder gegen Empfehlung unter Therapie
**Moxibustion (Moxa-Therapie, Moxen)**	–	Diskussion der vorliegenden Studien ohne Empfehlungsabgabe für oder wider	–	Bedingte Empfehlung (niedrige Evidenz) nach Therapie
**Phytotherapeutika und Nahrungsergänzungsmittel**				
**Ginseng**	Empfehlungsgrad 0: Empfehlung offen „kann“	Angabe einer doppelblinden RCT ohne signifikante Verbesserung der CRF, keine Empfehlung für oder wider	*Panax quinquefolius*: Das Gremium hat keinen Konsens erzielt	Bedingte Empfehlung: 2000 mg *Panax quinquefolius* täglich unter Therapie (niedrige Evidenz); keine ausreichende Evidenz für oder gegen Empfehlung nach Therapie
**Mistel**	Empfehlungsgrad 0: Empfehlung offen „kann“ für Lebensqualität	–	Das Gremium hat keinen Konsens erzielt	Keine ausreichende Evidenz für oder gegen Empfehlung nach Therapie
**Guarana-Trockenextrakt**	Empfehlungsgrad B: „sollte *nicht*“	Diskussion einer negativen RCT ohne Empfehlungsabgabe für oder wider	Empfehlung *gegen* eine Anwendung (II, D)	Keine ausreichende Evidenz für oder gegen Empfehlung
**Astragalus**	–	–	Empfehlung *gegen* eine Anwendung (II, D)	–
**Bryophyllum pinnatum**	Keine ausreichenden Daten für Empfehlung	–	–	–
**L‑Carnitin**	Empfehlungsgrad B: „sollte *nicht*“	Diskussion der vorliegenden Studien ohne Empfehlungsabgabe für oder wider	Empfehlung *gegen* eine Anwendung (II, D)	Empfehlung *gegen* eine Anwendung: (niedrige Evidenz)
**Coenzym Q**_**10**_	–	Diskussion der vorliegenden Studien ohne Empfehlungsabgabe für oder wider	Empfehlung *gegen* eine Anwendung (II, D)	Keine ausreichende Evidenz für oder gegen Empfehlung
**Zink**	Keine ausreichenden Daten für Empfehlung Endpunkt Prävention von Fatigue durch Zinksupplementation	–	–	–
**Pharmakologische Interventionen**				
**Kortikosteroide**	–	Kann erwogen werden für Patient:innen am Lebensende mit gleichzeitig bestehender Anorexie oder Schmerzen aufgrund von zerebralen oder ossären Metastasen	Mäßige Empfehlung für eine kurzzeitige Anwendung bei Patient:innen mit metastasierten Stadien (II, B)	Bedingte Empfehlung (niedriges Evidenzlevel)
**Psychostimulanzien** **(Modafinil/Armodafinil, Methylphenidat)**	–	Kann erwogen werden für Patient:innen unter Therapie und nach Abschluss der Therapie	Empfehlung *gegen* eine Anwendung: Modafinil/Armodafinil (II, D); das Gremium hat keinen Konsens erzielt: Methylphenidat, Dexmethylphenidat, lang wirksames Methylphenidat, Dexamphetamin	Empfehlung *gegen* eine Anwendung: (moderate Evidenz) unter und nach Therapie
**Antidepressiva**	–	Empfehlung *gegen* eine Anwendung	Empfehlung *gegen* eine Anwendung (II, D)	Empfehlung *gegen* eine Anwendung
**Donepezil**	–	–	Empfehlung *gegen* eine Anwendung (II, D)	–
**Eszopiclon, Megestrol Acetat, Melatonin**	–	Melatonin: Angabe einer doppelblinden RCT ohne signifikante Verbesserung der CRF, keine Empfehlung für oder wider	Empfehlung *gegen* eine Anwendung (II, D)	Melatonin: keine ausreichende Evidenz für oder gegen Empfehlung nach Abschluss der Therapie und am Lebensende
**Andere komplementärmedizinische Interventionen**				
**Anthroposophische Komplexbehandlung**	Empfehlungsgrad 0: Empfehlung „kann“ (nur für Brustkrebspatientinnen)	–	–	–
**(Ärztlich geleitetes) individualisiertes multimodales komplementärmedizinisches Therapieangebot**	Empfehlungsgrad 0: Empfehlung „kann“ (nur für Überlebende nach Brustkrebs)	–	–	–
**(Schwedische) Massagen**	Keine ausreichenden Daten für Empfehlung Endpunkt Reduktion von Fatigue und Ein- und Durchschlafstörungen	Empfehlung (Kategorie 1): für Patient:innen unter Therapie	–	Keine ausreichende Evidenz für oder gegen Empfehlung nach Therapie
**Lichttherapie**	–	Empfehlung (Kategorie 2A) für Patient:innen unter Therapie und nach Abschluss der Therapie	–	–
**Kunsttherapie**	Keine ausreichenden Daten für Empfehlung	–	–	–
**Bioenergiefeldtherapien**	Empfehlungsgrad B: „sollte *nicht*“	–	–	–

**ASCO American Society of Clinical Oncology**

**ASCO-SIO CRF:** Management of Fatigue in Adult Survivors of Cancer: ASCO-Society for Integrative Oncology Guideline Update. Mai 2024

– ASCO „starke Empfehlung“: „Bei Empfehlungen für eine Intervention überwiegen die erwünschten Wirkungen einer Intervention ihre unerwünschten Wirkungen. Alle oder fast alle informierten Personen würden die empfohlene Entscheidung für oder gegen eine Intervention treffen.“

– ASCO „bedingte Empfehlung (conditional)“: „Bei Empfehlungen für eine Intervention überwiegen die erwünschten Wirkungen wahrscheinlich die unerwünschten Wirkungen, aber es besteht eine beträchtliche Unsicherheit.“

*CRF* Cancer-related Fatigue

**ESMO European Society for Medical Oncology**

**ESMO CRF:** Cancer-related Fatigue: ESMO Clinical Practice Guidelines for diagnosis and treatment. März 2020

– ESMO I Evidenz aus mindestens einer ordnungsgemäß durchgeführten randomisierten kontrollierten Studie

– ESMO II Evidenz aus mindestens einer gut konzipierten klinischen Studie ohne Randomisierung; aus Kohorten- oder Fall-Kontroll-Analysestudien (vorzugsweise aus mehr als einem Zentrum); aus mehreren Zeitreihen oder aus eindrucksvollen Ergebnissen unkontrollierter Experimente

– ESMO B Die Autoren unterstützen die Empfehlung zur Anwendung mäßig

– ESMO C Die Autoren unterstützen die Empfehlung zur Anwendung geringfügig

– ESMO D Die Autoren unterstützen die Empfehlung gegen die Anwendung

**NCCN The National Comprehensive Cancer Network**

**NCCN CRF:** NCCN Guidelines for cancer related fatigue. Version 2.2024 – Oktober 2023

– NCCN Kategorie 1 Empfehlung: „Basierend auf Evidenz höherer Stufe besteht ein einheitlicher Konsens der NCCN, dass die Intervention angemessen ist.“

– NCCN Kategorie 2A Empfehlung: „Basierend auf Evidenz niedrigerer Stufe besteht ein einheitlicher Konsens der NCCN, dass die Intervention angemessen ist.“

*RCT* Randomized Controlled Trial

**S3-Leitlinie** Komplementärmedizin Onkologie: S3 Leitlinie Komplementärmedizin in der Behandlung von onkologischen Patienten. Version 2.0. Mai 2024

## Fazit

Fatigue bei Krebspatient:innen ist ein multidimensionaler Symptomkomplex, welcher eine interdisziplinäre und multimodale Herangehensweise erfordert. Phytotherapeutische Verordnungen, Akupunktur und Akupressur sowie Methoden aus der Mind-Body-Medizin erscheinen deshalb aufgrund ihres pleiotropen und selbstwirksamkeitsstärkenden Ansatzes vielversprechend. Insbesondere Interventionen, die Patient:innen darin unterstützen, tiefer in eine Entspannung kommen zu können, haben sich dabei als vorteilhaft erwiesen. Grundlage für eine Therapieentscheidung sollte, wie einleitend beschrieben, nicht nur die externe Evidenz sein, sondern auch die klinische Expertise des/der Behandler:in sowie die Patient:innenpräferenz.

Das Angebot eines Behandlungsversuchs sollte bei entsprechenden Beschwerden und Anliegen der Patient:innen immer gemacht werden, da eine ausreichende Symptomkontrolle die Lebensqualität wesentlich mitbedingt und darüber hinaus prognoserelevant sein kann. Die Auswahl der Behandlungsoptionen sollte dabei individuell auf die Gesamtsituation der Patient:innen abgestimmt werden. Dazu existieren inzwischen Leitlinien großer Fachgesellschaften, die Empfehlungen auf Basis des aktuellen und sich teils deutlich unterscheidenden Evidenzgrades geben und als Orientierungshilfe bei der Auswahl der geeigneten Methoden dienen können.

In der Phytotherapie haben bis heute nur Zubereitungen aus Ginsengwurzel im Kontext der CRF einen ausreichend hohen Evidenzgrad, wodurch diese bereits in die Leitlinienempfehlungen aufgenommen worden sind. Bei entsprechender vertiefter phytotherapeutischer Ausbildung des/der betreuenden Ärzt:in und diesbezüglich sorgfältig durchgeführter Fallaufnahme des/der Patient:in ergeben sich jedoch weiterführende Therapieoptionen, die hier nur angedeutet werden konnten.

Somit liegen zum aktuellen Zeitpunkt für verschiedene komplementärmedizinische Therapieverfahren zum Teil Belege für, zum Teil zumindest Hinweise auf eine klinische Wirksamkeit bei dem Symptomkomplex der krebsbedingten Fatigue vor. Diese Verfahren können in dieser Indikation, die für viele Patient:innen mit einem hohen Leidensdruck verbunden ist, hilfreich eingesetzt werden. Es ist bedauerlich, dass die Kosten für keines der genannten Verfahren regelhaft von den gesetzlichen Krankenversicherungen übernommen werden. Vor diesem Hintergrund sind sie leider ausschließlich Personen vorbehalten, die finanziell in der Lage sind, die anfallenden Kosten selbst zu tragen. Die steigende Verfügbarkeit von web- oder App-basierten Programmen könnte dazu beitragen, diesem Problem entgegenzuwirken. Besonders hinsichtlich Kosteneffizienz, Skalierbarkeit und einer barrierefreien Verfügbarkeit – auch außerhalb von Ballungsgebieten, in denen vereinzelt bereits komplexe Interventionen aus der Mind-Body-Medizin im Rahmen klinischer und tagesklinischer Programme angeboten werden – wären solche digitalen Lösungen für viele interessierte Patient:innen von hohem Interesse.
